# Association between depression symptoms and cognitive frailty in chronic kidney disease patients: a cross-sectional study

**DOI:** 10.3389/fpsyt.2025.1648424

**Published:** 2025-09-24

**Authors:** Peng Zhang, Aiqing Fan, Nian Xie, Jing Jiang, Shuzhi Peng

**Affiliations:** ^1^ School of Public Health, Hainan Medical University, Haikou, China; ^2^ School of Clinical Medicine, Shanghai University of Medicine and Health Sciences, Shanghai, China; ^3^ General Medical Department, Shanghai Pudong New Area Sunqiao Community Health Service Center, Shanghai, China; ^4^ School of Engineering, China Pharmaceutical University, Nanjing, China; ^5^ Postdoctoral Research Workstation, State Information Center (SIC), Beijing, China; ^6^ College of Health Management, Shanghai Jian Qiao University, Shanghai, China

**Keywords:** chronic kidney disease, depression symptoms, cognitive frailty, CES-D, RCS

## Abstract

**Objective:**

Cognitive frailty (CF) and depressive symptoms are prevalent in patients with chronic kidney disease (CKD) and may synergistically exacerbate adverse health outcomes. This study examined their association to inform early intervention strategies.

**Methods:**

Demographic and clinical data were collected from CKD patients across three hospitals in Shanghai. Depressive symptoms and CF status were assessed via standardized questionnaires. A logistic regression model and restricted cubic spline (RCS) analysis were employed to evaluate the association between depressive symptoms and CF.

**Results:**

CF was diagnosed using the Frailty Phenotype (FP), Montreal Cognitive Assessment (MoCA), and Clinical Dementia Rating (CDR). Among 800 participants, 317 exhibited CF (prevalence: 39.6%). The adjusted logistic regression model revealed a significant positive association between Center for Epidemiologic Studies Depression (CES-D) scores and CF (OR=1.124, 95% *CI*: 1.094–1.15*6, p*<0.001). RCS analysis demonstrated a nonlinear dose-response relationship: CF prevalence increased with rising CES-D scores until plateauing at a score of 9. The odds ratio (OR) exceeded the statistical significance threshold when CES-D scores reached 12. Subgroup analyses consistently supported this dose-response pattern.

**Conclusion:**

Depressive symptoms are significantly associated with CF in CKD patients. Routine CES-D screening and provision of psychological support for patients scoring ≥12 may mitigate CF risk.

## Introduction

In recent years, the prevalence of CKD has increased year by year, and the prevalence rate of the general population in the world has reached 14.3% (1). Recent studies show that the incidence of CF in patients with CKD is as high as 58.8% (2), which may have an important impact on patients’ health and reduce their compliance with treatment plans and quality of life. The factors related to cognitive weakness include female, 75 years old and above, poor economic status, rural residence, irregular exercise and cerebrovascular disease, etc. Primary health care is an important way to identify cognitive impairment early (3).

Now research has confirmed that CF can accelerate the development of patients’ physical frailty and cognitive impairment, seriously affect patients’ daily life, activity ability and quality of life (4), and is also an important predictor of serious adverse health outcomes such as falls, dementia, disability and even death (5). The increase of body toxins in CKD patients will lead to physical frailty and cognitive function decline (6). The coexistence of the two will aggravate the decline of patients’ self-management ability and medication compliance, and even develop into dementia, thus aggravating the progress of the course of CKD patients (7).

With the continuous progress of the course of CKD, the probability of depressive symptoms is higher due to abnormal metabolism, hemodynamic changes and vascular diseases, toxin accumulation and energy consumption caused by long-term dialysis treatment (8). Ma et al. investigated non-demented elderly people and found that the prevalence of CF in depressed people reached 15.1% (9). Their research results showed that depression symptoms were an independent related factor of CF in healthy people. Many studies have confirmed that depressive symptoms are an important risk factor for CF (10). There is a similar pathological mechanism between depression symptoms and CF. Persistent depressive symptoms will reduce the desire of CKD patients to participate in social activities, thus reducing their social support and greatly increasing the risk of CF (11). Therefore, it is very important to study the relationship between depressive symptoms and CF in CKD patients.

Therefore, in our study, we studied the potential correlation between CES-D score and CF, with the aim of better controlling CF by controlling the occurrence of depressive symptoms in CKD patients. In the past, many studies focused on the relationship between depression symptoms and CF (12), but all of them were included in the model for analysis as classified variables, which would lose some information as continuous variables. At present, there are few studies on the relationship between CES-D score as a continuous variable and the risk of CF (13). RCS can intuitively describe the nonlinear relationship between continuous variables and outcomes, and has been applied by more and more researchers. This study investigates the association between CES-D scores and CF in CKD patients using restricted cubic spline (RCS) analysis to characterize nonlinear relationships.

## Materials and methods

### Study population and design

**Figure 1:**
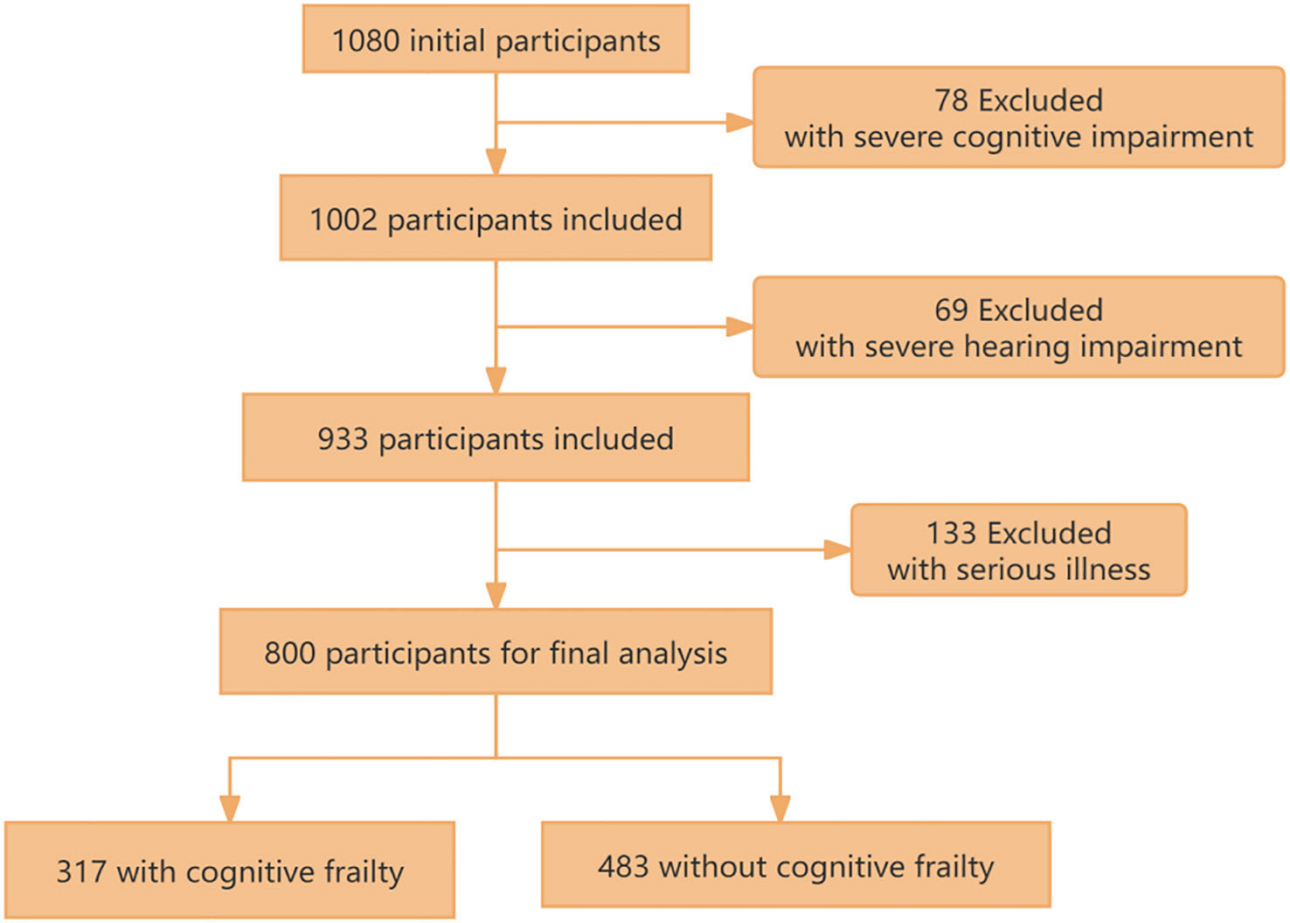
The flow chart of study population selection.

The study cohort was derived from three tertiary hospitals in Shanghai. Potential chronic kidney disease (CKD) participants were initially identified through systematic screening of electronic medical records. Following rigorous eligibility assessment, 280 participants were excluded based on predefined exclusion criteria (presence of severe cognitive/hearing impairment or critical illness status). The remaining 800 participants (73.6% of initially screened candidates) provided written informed consent, achieving a final participation rate of 85.2% among medically qualified candidates (**Figure 1**).

### Measurement methods

Depression symptoms and CF were assessed using validated scales. Depression symptoms were assessed by the CES-D, which was compiled by Radloff and others of the National Institute of Mental Health (NIMH) (14). The CES-D scale exhibits validated reliability in chronic kidney disease populations (Cronbach’s α=0.85) (15).

The diagnostic criteria for CF were established in accordance with the 2013 assessment standards promulgated by the International Academy on Nutrition and Aging (IANA) and the International Association of Gerontology and Geriatrics (IAGG) (16). Participants were required to meet the following concurrent criteria: (1) FP scale scores ranging from 3 to 5; (2) MoCA scale score <26; (3) Absence of dementia diagnosis with CDR scale score of 0.5 (17).

Frailty Phenotype (FP), proposed by Freid et al. in 2001 (18), includes five phenotypes: unexplained weight loss, self-incriminating fatigue, slow walking speed, weak grip strength, and less physical activity. Scores range 0-5; ≥3 indicates frailty, 1–2 pre-frailty, 0 no frailty, with higher scores indicating greater severity.

Montreal Cognitive Assessment (MoCA), developed by Nasreddine et al. in 2004 (19), assesses seven domains: visuospatial ability, naming, attention, language, abstract reasoning, memory (immediate and delayed), and computation/spatial orientation. Scores range 0-30; ≥26 indicates normal cognition, <26 suggests impairment, with a 1-point addition for education below 12 years.

Clinical Dementia Rating (CDR): Developed by Hughes et al., the CDR (20)scale covers six domains: problem-solving, orientation, memory, social activities, family/work/hobbies, and self-care. Evaluated by trained clinical staff, scores are synthesized from sub-items, not summed. Scores indicate: 0 for normal cognition, 0.5 for questionable dementia, 1.0 for mild, 2.0 for moderate, and 3.0 for severe dementia.

In our study, we employed three primary assessment tools: the CES-D, FP, MoCA, and CDR, all administered by experienced clinical physicians. These scales are widely used in China (21).

### Covariate assessment

Covariate evaluation includes social demography, disease-related situation and lifestyle characteristics. The selection of these covariates was based on their proven association with cognitive function in prior studies and their strong correlation with the demographic characteristics of our target population (22).

Socio-demographic characteristics: gender, age, BMI (kg/m², categorized as <18.5, 18.5-24, or ≥24), marital status (married, never married, widely/divided), education level (below high school, high school, college or above), living conditions (alone, with family, with caregivers) (23). Disease-related conditions: course of CKD, dialysis, hypertension, diabetes, heart disease. Lifestyle characteristics: smoking, drinking, exercise, society. Smoking/drinking criteria: Never: <6 months or never; Current: >6 months and current within 30 days (24); Former: >6 months but not current. Exercise: one session defined as >50 min moderate (e.g., walking, Tai Chi) or >25 min high intensity (e.g., running, swimming, climbing, ball games, square dancing (25)).

### Statistical analysis

Study participants were stratified into two cohorts based on the presence or absence of CF. In the analysis of weighted features, continuous variables were expressed as mean ± standard error (SE), while categorical variables were reported as percentages.

Statistical comparisons of baseline characteristics were performed using t-test for continuous variables and chi-square test for categorical variables. The association between depressive symptoms and CF was quantified through multivariable logistic regression analysis, with results expressed as adjusted odds ratios (OR) and corresponding 95% confidence intervals (CI) (26). In addition, we also conducted subgroup analysis to investigate whether this association was changed by social demography, disease-related conditions or lifestyle characteristics in the fully adjusted model (27). A two-segment linear regression model was subsequently constructed to determine the inflection point. All statistical analyses were performed using R software (version 4.3.3), with P-values<0.05 considered statistically significant.

## Results

The basic characteristics of 800 participants, of whom 317 (39.6%) have CF. The average CES-D score of our population is 14.91. Among all the participants, 482 (60.3%) were male, with an average age of 48.23 years, and 470 (58.8%) had a below high school education. Compared with participants without CF participants with CF have higher CES-D scores. There are differences in CF between CKD patients with HD and those without HD, and the difference between drinking and CF in lifestyle characteristics is statistically significant. (See **Table 1** for details.)

**Table 1:** Weighted characteristics of participants in this study by CF.

Variable	All participants	Non-CF	CF	P-value
Sex				0.461
Male	482 (60.3%)	296 (37.0%)	186 (37.0%)	
Female	318 (39.8%)	187 (23.4%)	131 (16.4%)	
Age	48.23 ± 8.030	47.86 ± 8.444	48.79 ± 7.331	<0.001
BMI(kg/m^2)^				0.495
<18.5	106 (13.3%)	61 (7.6%)	45 (5.6%)	
18.5-24	374 (46.8%)	221 (27.6%)	153 (19.1%)	
>24	320 (40.0%)	201 (25.1%)	119 (14.9%)	
Marital status				
Married	386 (48.3%)	226 (28.3%)	160 (20.0%)	0.588
Never married	180 (22.5%)	111 (13.9%)	69 (8.6%)	
Widowed/divorced	234 (29.3%)	146 (18.3%)	88 (11.0%)	
Education attainment				0.556
Below high school	470 (58.8%)	277 (34.6%)	193(24.1%)	
High school	194 (24.3%)	123 (15.4%)	71 (8.9%)	
College or above	136 (17.0%)	83 (10.4%)	53 (6.6%)	
Living conditions				0.557
Living alone	115 (14.4%)	71 (8.9%)	44 (5.5%)	
Live with family	541 (67.6%)	320 (40.0%)	221(27.6%)	
Living with caregivers	144 (18.0%)	92 (11.5%)	52 (6.5%)	
Course				0.697
CKD1	76 (9.5%)	50 (6.3%)	26 (3.3%)	
CKD2	194 (24.3%)	115 (14.4%)	79 (9.9%)	
CKD3	272 (34.0%)	158 (19.8%)	114 (14.3%)	
CKD4	132 (16.5%)	84 (10.5%)	48 (6.0%)	
CKD5	126 (15.8%)	76 (9.5%)	50 (6.3%)	
Dialysis				0.635
No	470 (58.8%)	287 (35.9%)	183(22.9%)	
Yes	330 (41.3%)	196 (24.5%)	134(16.8%)	
Hypertension				0.053
No	308 (38.5%)	199 (24.9%)	109(13.6%)	
Yes	492 (61.5%)	284 (35.5%)	208(26.0%)	
Diabetes				0.058
No	333 (41.6%)	214 (26.8%)	119 (14.9%)	
Yes	467 (58.4%)	269 (33.6%)	198(24.8%)	
HD				0.003
No	687 (85.9%)	429 (53.6%)	258(32.3%)	
Yes	113 (14.1%)	54 (6.8%)	59 (7.4%)	
Smoking				0.333
Never smoking	463 (57.9%)	278 (34.8%)	185 (23.1%)	
Current smoking	235 (29.4%)	149 (18.6%)	86 (10.8%)	
Former smoking	102 (12.8%)	56 (7.0%)	46 (5.8%)	
Drinking				0.019
Never drinking	459 (57.4%)	277 (34.6%)	182(22.8%)	
Current drinking	239 (29.9%)	156 (19.5%)	83 (10.4%)	
Former drinking	102 (12.8%)	50 (6.3%)	52 (6.5%)	
Exercise				0.938
Every day	152 (19.0%)	92 (11.5%)	60 (7.5%)	
5–6 times a week	175 (21.9%)	108 (13.5%)	67 (8.4%)	
3–4 times a week	316 (39.5%)	189 (23.6%)	127(15.9%)	
1–2 times a week	104 (13.0%)	60 (7.5%)	44 (5.5%)	
Never	53 (6.6%)	34 (4.3%)	19 (2.4%)	
Society				0.773
Every day	120 (15.0%)	73 (9.1%)	47 (5.9%)	
5–6 times a week	157 (19.6%)	100 (12.5%)	57 (7.1%)	
3–4 times a week	312 (39.0%)	187 (23.4%)	125(15.6%)	
1–2 times a week	164 (20.5%)	98 (12.3%)	66 (8.3%)	
Never	47 (5.9%)	25 (3.1%)	22 (2.8%)	
Depressive symptoms	14.910 ± 6.086	13.360 ± 5.260	17.260 ± 6.497	<0.001

Mean ± SE for continuous variables and % for categorical variables. HD, heart disease.

**Figure 2:**
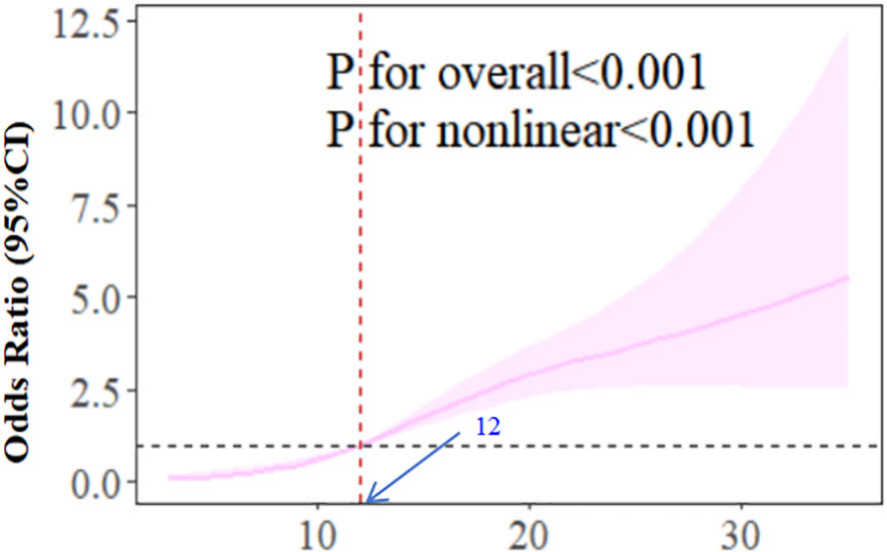
The dose-response relationship between CES-D score and CF.

**Table 2** shows CES-D score significantly related to CF, with positive OR associations across all models (OR≈1.12, *p*<0.001). Due to this relationship, we performed piecewise regression (**Table 3**), identifying an inflection point at CES-D=9: CF prevalence increases until CES-D=9, then OR plateaus. We adopted a cubic spline model based on model 3. **Figure 2** indicates OR increases with CES-D score, becoming significantly >1 at CES-D=12.

**Table 2:** Logistic regression results of associations between depression and CF.

Model	OR	95%*CI*	P-value
Model 1			
CES-D score	1.118	(1.089, 1.147)	<0.001
Model 2			
CES-D score	1.120	(1.091, 1.150)	<0.001
Model 3			
CES-D score	1.124	(1.094, 1.156)	<0.001

Model 1, no covariate was adjusted. Model 2, sex, age, BMI, marital status, education attainment, living conditions were adjusted. Model 3, sex, age, BMI, marital status, education attainment, living conditions, course, dialysis, hypertension, diabetes, HD, smoking, drinking, exercise, society were adjusted.

**Table 3:** Threshold effect analysis between depression and CF.

Outcome	OR (95% *CI*)	P-value
Two-piecewise linear regression model		
CES-D<9	6.466 (2.441-26.547)	0.002
CES-D≥9	1.103 (1.072-1.135)	<0.001
Log-likelihood ratio test		<0.001

Sex, age, BMI, marital status, education attainment, living conditions, course, dialysis, hypertension, diabetes, HD, smoking, drinking, exercise, society were adjusted in the threshold effect analysis.

## Discussions

### The status of depression symptoms and CF

There are 800 participants in this study, of which 317 (39.6%) have CF. The incidence of CF is higher than that of Chang (28) and others. The possible reason is that the object of this study is CKD patients treated in hospitals. The hospital environment will make patients more prone to CF. Therefore, we should strengthen the identification of CF in hospitalized CKD patients and explore effective CF intervention programs. The average CES-D score of our survey population is 14.91, and 140 people (17.5%) have a score of ≥20, which means that 17.5% of the 800 CKD patients surveyed in this study have depressive symptoms. Affected by the disease, the patient’s physical function is weakened, which leads to the occurrence of negative psychological emotions such as depression symptoms (29). Compared with participants without CF, participants with CF have higher average CES-D scores. As far as pathophysiological mechanism is concerned, CF and depression symptoms are interrelated, and there are some similar risk factors between them, such as cerebrovascular disease, chronic inflammation and oxidative stress (30).

### Relationship between depression symptoms and CF

The RCS results of model 3 show that with the increase of CES-D score, the OR of correlation between CES-D and CF increases. When the CES-D score reaches 12, the OR is significantly higher than 1. This suggests that when the CES-D score is higher than 12, we should pay attention to the patients’ depression symptoms and give them psychological counseling, instead of waiting until the depression symptoms score is ≥20 to pay attention to the patients’ depression symptoms.

The dose-response curve derived from restricted cubic spline analysis further corroborates the non-linear association, demonstrating a steeper slope in CF risk elevation below the CES-D threshold of 9 compared to the gradual increase observed beyond this inflection point. This pattern persists even after comprehensive adjustment for sociodemographic factors (age, marital status, education), lifestyle variables (smoking, alcohol consumption, exercise frequency), and clinical characteristics (dialysis status, hypertension control, diabetes management). The consistency across analytical approaches—from crude models to fully adjusted threshold regression—strengthens the robustness of our findings.

Notably, the identified threshold (CES-D=9) precedes conventional clinical thresholds for depression symptoms diagnosis (typically ≥16), suggesting subclinical depressive symptoms may already exert measurable impacts on cognitive function in CKD populations. This observation aligns with emerging evidence highlighting the continuum of depressive symptomatology in chronic disease management (31, 32). Pathophysiologically, this association might be mediated through shared mechanisms including chronic inflammation (elevated CRP and IL-6 levels), hypothalamic-pituitary-adrenal axis dysregulation, and cerebral microvascular changes—all documented in both depression symptoms and cognitive impairment pathways (33, 34).

Our findings extend previous work by Chang et al. through quantitative characterization of the exposure-response relationship (33, 34). The 12% increased CF risk per unit CES-D increment (Model 3 OR=1.124, 95%CI 1.094-1.156) exceeds estimates from general population studies, potentially reflecting disease-specific vulnerability in CKD patients (35). The persistent association across progressively adjusted models argues against complete confounding by measured covariates, though residual confounding from unmeasured factors (e.g., apolipoprotein E genotype, sleep architecture disturbances) remains possible (33–35).

### Strengths and limitations

CF is a reversible or potentially reversible heterogeneous clinical syndrome. Early recognition and effective intervention of CF in CKD patients can greatly help to reverse the occurrence of CF. The findings of this study may be valuable for CF. It provides health professionals with additional insights on the close relationship between depression symptoms and CF. Because of the cross-sectional study design, we can’t infer causal reasoning or exclude two-way relationship, and there is a certain selection bias in variable selection.

Future studies will conduct further exploration through prospective cohort studies. The assessment of depressive symptoms in this investigation relied on self-report questionnaires, representing a subjective rather than objective measurement approach. Consequently, the sample size derived from the research data is constrained, being limited to three healthcare institutions. Subsequent multi-center investigations would be beneficial to mitigate potential result deviations (36). Notwithstanding these limitations, the present analysis yields significant findings that provide a foundation for further research.

## Conclusion

Our study found that there was a nonlinear correlation between CES-D score and CF in CKD patients after adjusting for potential confounding factors. depression symptoms were considered to be an important influencing factor of CF, and there is a threshold effect in the above association. Health professionals can regard this association as an integral part of health care evaluation and management of patients with CF. In addition, the evidence of this study also encourages the general population to carry out regular self-rating depression symptoms, so as to maintain good mental health and reduce the occurrence of CF.

## Data Availability

The original contributions presented in the study are included in the article/supplementary material. Further inquiries can be directed to the corresponding author.
